# Systemic Inflammatory Burden Correlates with Severity and Predicts Outcomes in Patients with Cardiogenic Shock Supported by a Percutaneous Mechanical Assist Device

**DOI:** 10.1007/s12265-020-10078-5

**Published:** 2020-10-19

**Authors:** Nikolaos A. Diakos, Katherine Thayer, Lija Swain, Maithri Goud, Pankaj Jain, Navin K. Kapur

**Affiliations:** 1 Division of Cardiology, Department of Internal Medicine, Tufts Medical Center, Boston, MA USA; 2 Department of Medicine/Division of Cardiology, The Cardiovascular Center for Research and Innovation (CVCRI), Acute Circulatory Support Program, Interventional Research Laboratories, Molecular Cardiology Research Institute, Tufts Medical Center, Boston, MA USA

**Keywords:** Cardiogenic shock, Inflammation, Mechanical circulatory support, Heart failure

## Abstract

In-hospital mortality associated with cardiogenic shock (CS) remains high despite introduction of mechanical circulatory support. In this study, we aimed to investigate whether systemic inflammation is associated with clinical outcomes in CS. We retrospectively analyzed systemic cytokine levels and the neutrophil-to-lymphocyte ratio (NLR), a marker of low-grade inflammation, among 134 patients with CS supported by VA-ECMO or Impella. Sixty-one percent of patients survived CS and either underwent device explantation or were bridged to LVAD or cardiac transplant. IL6 was the predominant circulating cytokine. IL6 levels were reduced after circulatory support in survivors. NLR pre-device implantation was significantly lower in patients with earlier stages of cardiogenic shock. Compared with non-survivors, survivors had a lower pre-device NLR and NLR was independently predictive of survival after adjusting for other covariates. In summary, NLR is a widely available marker of inflammation and correlates with in-hospital mortality among patients with cardiogenic shock requiring percutaneous mechanical circulatory support.

**Electronic supplementary material:**

The online version of this article (10.1007/s12265-020-10078-5) contains supplementary material, which is available to authorized users.

**Graphical Abstract:**
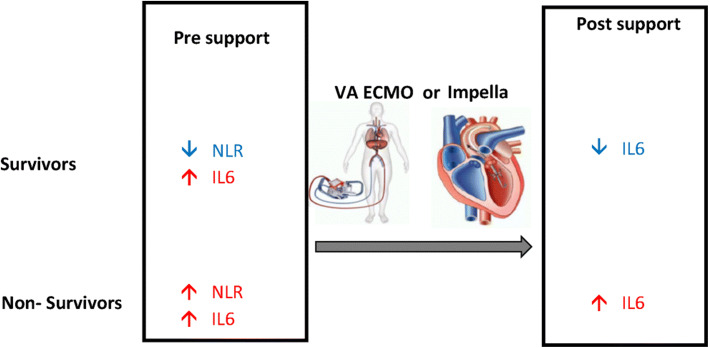
Survivors present with lower NLR levels prior to percutaneous device implantation. Both survivors and non survivors present with elevated IL6 levels. IL6 levels decrease after percutaneous support (ECMO or Impella) only in survivors and continue to rise in non-survivors.

## Introduction

Despite the introduction of acute mechanical circulatory support, the mortality from cardiogenic shock (CS) remains as high as 50% [1]. Inflammation plays a central role in the pathogenesis of acute and chronic heart failure (HF) and prior studies have shown a correlation between systemic inflammation and adverse HF outcomes [2, 3]. The development of novel anti-inflammatory agents with promising results in the treatment of cardiovascular disease underlines the necessity to further investigate the role of inflammation as a prognostic marker and as a therapeutic target for the management of CS [4].

Neutrophil-to-lymphocyte ratio (NLR) has been proposed as a surrogate marker of systemic inflammation. Increased NLR is associated with systemic inflammatory response syndrome (SIRS) in the setting of bacterial infection, burn injury, pancreatitis, and acute liver failure [5–7], and prior studies have demonstrated the role of NLR as a predictor of outcomes for critically ill patients [7].

In this retrospective analysis, we aimed to investigate whether systemic inflammatory burden correlates with the severity of CS and whether NLR could serve as an independent predictor of survival.

## Methods

We retrospectively studied 134 patients who were hospitalized with CS and supported by a temporary percutaneous assist device (extra-corporeal membrane oxygenator (ECMO) or Impella) at Tufts Medical Center between July 2012 and August 2019. Patients with post-cardiotomy shock or patients with septic shock were excluded from the study. Laboratory data, clinical, echocardiographic and hemodynamic data were collected within and up to 24 h prior to device implantation. Additionally, blood samples were collected at the time of device implantation and 72 h later, while the patient was on circulatory support to assess cytokine levels in either serum or plasma. The blood collection was approved by the IRB and the study subjects provided informed consent prior to sample collection. The collection tube was immediately placed on ice and the sample was subsequently centrifuged at 2000 g for 12 min for serum οr plasma collection. The serum or plasma was aliquoted in Eppendorf tubes and was stored at − 80 °C. Interleukin 6 (IL6), tumor necrosis factor-a (TNFa), and interferon-g (IFNg) were measured using ELISA kits (R&D systems, Minneapolis).

We defined outcomes by survival at discharge and CS severity. *Survivors* as the subgroup of patients who survived CS to hospital discharge and either underwent device explantation or were bridged to a left ventricular assist device (LVAD) or cardiac transplant. The rest of the patients were defined as *non-survivors*. The severity of CS was defined based on the recently published Society for Cardiovascular Angiography and Interventions (SCAI) shock classification and patients were classified based on the most severe stage reached over their in-patient stay [8].

### Statistical Analysis

For descriptive analysis of survivors and non-survivors, categorical variables were presented as frequencies and percentages and were compared using the Pearson chi-square test or the Fisher’s exact test when appropriate. Continuous variables were presented as mean ± SEM and were compared using independent samples *t* tests. Welch correction was used for values with unequal variances. NLR was used as a marker of inflammation and compared between survivors and non-survivors; between SCAI C, SCAI D, and SCAI E patients; and between patients with ischemic and non-ischemic cardiomyopathy using independent sample *t* test. Paired samples *t* test was used for NLR comparisons before and after circulatory support. Univariate logistic regression was used to identify predictors of in-hospital mortality, including NLR and other pre-implant clinical, hemodynamic, and laboratory variables. A multivariable logistic regression model was built to assess NLR’s independent prediction of in-hospital mortality by adjusting for significant predictors identified by univariate analysis. Additionally, the area under receiver operating characteristic (ROC) curve for in-hospital mortality was calculated in order to evaluate the predictive performance of NLR.

Exploratory analyses were also performed to assess changes in pre- and post-support cytokine levels. Pre- and post-support IL6, TNFa, and IFNg were evaluated as markers of inflammation and compared using paired samples *t* test. This analysis of IL6 was then stratified by Impella and ECMO subgroups. Percent change in IL6 from pre- to post-support was also compared between survivors and non-survivors using independent sample *t* test.

Significance for all statistical tests was determined using an alpha level of 0.05.

## Results

### Study Population

The study included patients presenting with CS requiring support by ECMO or Impella. Among 134 patients admitted with CS between July 2012 and August 2019, 34 patients with post-cardiotomy shock or concomitant septic shock were excluded from our analysis. Out of the remaining 100 patients, 12 reached a maximum SCAI class of C (12%), 82 reached class D (82%), and 6 reached class E (6%) (Online resource 1). Sixty-one patients survived (61%) and either underwent device explantation (*n* = 41) or were bridged to LVAD (*n* = 16) or cardiac transplant (*n* = 4). Baseline clinical characteristics of survivors and non-survivors are presented in Table 1. Sixty-three patients were supported by Impella and 37 were supported primarily by ECMO. Out of the 37 patients supported by ECMO, four patients had Impella implantation and two had IABP implantation for LV venting. Compared with non-survivors, survivors were younger (54 ± 2.0 vs 63 ± 1.5 years, *p* = 0.0012), had better renal function (creatinine: 1.8 ± 0.1 vs 2.5 ± 0.2 mg/dl, *p* = 0.002), higher Hgb (12 ± 0.3 vs 10 ± 0.4 g/dl, *p* = 0.0012), lower red cell distribution width (RDW) (14.8 ± 0.3 vs 16.6 ± 0.5, *p* = 0.0006), and lower right atrial pressure (15 ± 0.8 vs 19 ± 1.0 mmHg, *p* = 0.004) at the time of device implantation. Also, survivors were less likely to have history of chronic kidney disease and diabetes **(**Table 1**).**

**Table 1:** Baseline clinical and hemodynamic characteristics of the study population before device implantation

	Survivors (*n* = 61)	Non-survivors (*n* = 39)	*p* value
Men, *n* (%)	42 (69%)	28 (72%)	0.8
Age	54 ± 2	63 ± 1.5	0.0012*
Etiology, *n* (%)			
Ischemic	42 (69%)	31 (80%)	0.2
Non-ischemic	19 (31%)	8 (20%)	
Chronicity of heart failure, *n* (%)			
Acute heart failure	32 (52%)	17 (44%)	0.4
Acute on chronic heart failure	29 (48%)	22 (56%)	
Device, *n* (%)			
ECMO	23 (62%)	14 (38%)	0.9
Impella	38 (60%)	25 (40%)	
Hypertension, *n* (%)	31 (51%)	25 (64%)	0.2
Diabetes mellitus, *n* (%)	16 (26%)	18 (46%)	0.04*
Chronic kidney disease, *n* (%)	13 (21%)	16 (41%)	0.04*
Serum concentrations			
Creatinine, mg/dL	1.8 ± 0.1	2.5 ± 0.2	0.002*
Sodium, mmol/L	136 ± 0.8	136 ± 0.9	0.6
Hemoglobin, g/dL	12 ± 0.3	10 ± 0.4	0.0012*
RDW	14.8 ± 0.3	16.6 ± 0.5	0.0006*
AST, mg/dl	1029 ± 404	751 ± 250	0.5
ALT, mg/dl	526 ± 175	556 ± 179	0.8
ALP, mg/dl	89 ± 7	87 ± 7	0.6
Total bilirubin, mg/dl	1.5 ± 0.16	2.2 ± 0.33	0.2
Cardiac catheterization			
Mean right atrial (mmHg)	15 ± 0.8	19 ± 1	0.004*
Pulmonary capillary wedge (mmHg)	24 ± 1	25 ± 1	0.8
Systolic pulmonary arterial (mmHg)	44 ± 2	49 ± 3	0.1
Diastolic pulmonary arterial (mmHg)	24 ± 1	25 ± 1	0.8
Cardiac output	3.9 ± 0.2	4 ± 0.2	0.6
Echocardiography			
LVEF (%)	16 ± 1	18 ± 2	0.5
LVEDD (cm)	5.6 ± 0.4	5.8 ± 03	0.7
LVESD (cm)	4.9 ± 0.4	4.8 ± 0.4	0.8
Mechanical ventilation	45 (74%)	30 (77%)	0.8

Comparison of baseline demographic, laboratory, hemodynamic, and echocardiographic parameters between survivors and non-survivors. *RDW* red cell distribution width, *LVEDD* left ventricular end diastolic diameter, *LVEF* left ventricular ejection fraction, *LVESD* left ventricular end systolic diameter, *AST* aspartate transaminase, *ALT* alanine transaminase, *ALP* alkaline phosphatase. *p < 0.05

### Circulating Cytokines in Patients with Cardiogenic Shock

**Fig. 1:**
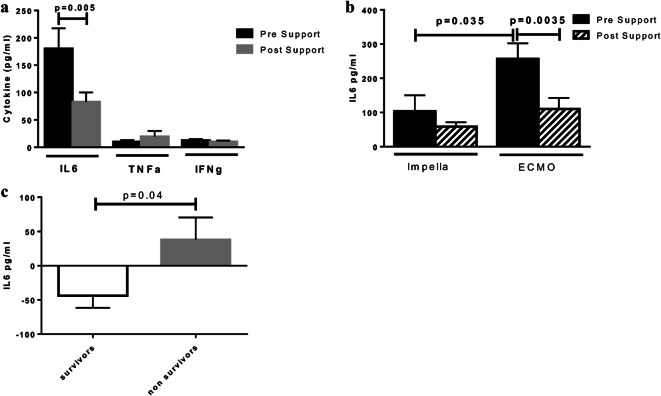
**a** Circulating cytokine levels before percutaneous device implantation and 72 h after implantation. **b** Circulating IL6 levels in patients supported by Impella or ECMO at the time of device implantation and 72 h after implantation. Significantly higher levels of IL6 are detected in patients with biventricular failure requiring ECMO support compared with patients with isolated LV failure requiring Impella support. **c** Comparison of percentile change of circulating IL6 after percutaneous mechanical support between survivors and non-survivors. There is reduction of IL6 in survivors, while IL6 continues to rise in non-survivors. IL6, Interleukin 6; TNFa, tumor necrosis factor alpha; INFg, interferon gamma; ECMO, extra-corporeal membrane oxygenator

IL6 was the predominant circulating cytokine and it was detected in high levels in the serum of patients with cardiogenic shock (Fig. 1a). On the contrary, TNFa and IFNg were detected in very low levels (Fig. 1a). Patients with non-ischemic cardiomyopathy had higher IL6 and TNFa levels prior to device implantation (Online resource 2). There was no difference in the levels of IFNg between ischemic and non-ischemic cardiomyopathy. Percutaneous mechanical circulatory support led to significant reduction of IL6 (180.3 ± 37.2 vs 82.7 ± 17.3 pg/ml, *p* = 0.005) while TNFa (9.8 ± 3.5 vs 19.5 ± 10.2 pg/ml, *p* = 0.3) and IFNg (12.5 ± 2.7 vs 10.2 ± 1.8 pg/ml, *p* = 0.9) levels remained unchanged (Fig. 1a). Patients requiring ECMO support had higher levels of IL6 at the time of device implantation compared with patients supported by Impella (256.7 ± 45.8 vs 103.9 ± 46.5 pg/ml, *p* = 0.035) (Fig. 1b). ECMO support for 72 h resulted in significant reduction of circulating IL6 levels (256.7 ± 45.8 vs 110.6 ± 32 pg/ml, *p* = 0.0035) (Fig. 1b). Survivors exhibited significant reduction in IL6 serum levels while IL6 levels in non-survivors continued to rise after mechanical support (− 43.8 ± 17.6% vs 37.9 ± 32.6%, *p* = 0.04) (Fig. 1c).

### Correlation of NLR with Disease Severity and Mortality

**Fig. 2:**
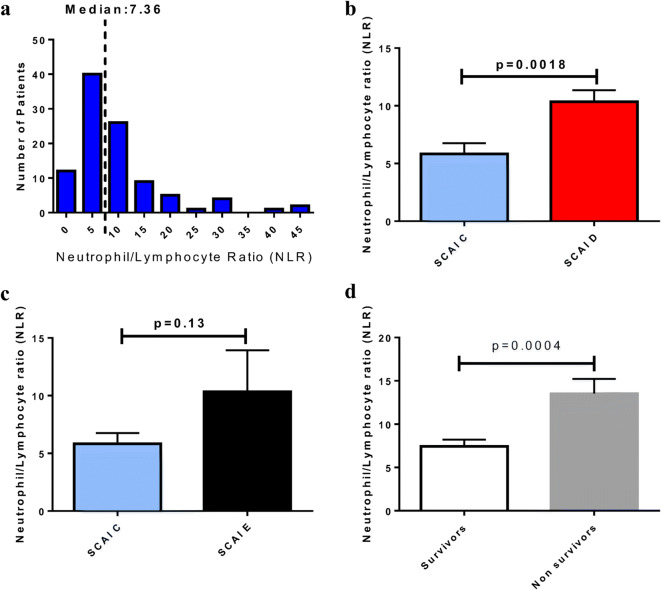
**a** Distribution of NLR prior to percutaneous device implantation. **b** Lower NLR levels in patients with CS SCAI class C compared with SCAI class D. **c** Non-significant difference in NLR levels between patients with CS SCAI class C and E. (d) Lower NLR levels in survivors compared with non-survivors at the time of percutaneous device implantation. CS, cardiogenic shock; NLR, neutrophil-to-lymphocyte ratio

White blood cell (WBC) populations were measured prior to device implantation. Total WBC and neutrophils were lower while lymphocytes were higher in survivors compared with non-survivors (Online resource 3). The median NLR prior to percutaneous device implantation was 7.36 (Fig. 2a). The NLR was significantly lower in patients with SCAI class C compared with class D suggesting lower systemic inflammatory burden in patients at earlier stages of CS (5.8 ± 0.9 vs 10.4 ± 1, *p* = 0.0018) (Fig. 2b). NLR also tended to be higher in SCAI class E group when compared with SCAI class C but the difference was not statistically significant (Fig. 2c). Survivors also had lower NLR levels at the time of temporary support device implantation compared with non-survivors (7.4 ± 0.8 vs 13.5 ± 1.7, *p* = 0.0004) (Fig. 2d).

There was no difference in baseline NLR levels when comparing patients with cardiogenic shock and ischemic cardiomyopathy to patients with cardiogenic shock and non-ischemic cardiomyopathy (Online resource 2).

Patients with evidence of infection at presentation (i.e., pneumonia, bacteremia, sepsis) were excluded from our study. During the period of percutaneous mechanical circulatory support, thirty-five of the patients had clinical findings that were suspicious for infection and they were covered empirically with antibiotics. The threshold for initiation of antibiotics was low, especially for patients supported by ECMO. These patients did not necessarily have positive cultures. We compared the NLR at baseline and there was no difference between the patients with suspected infection and those without suspected infection (Online resource 4). In both groups, NLR increased after mechanical support; however, it reached statistical significance only in the group without suspected infection. There was no difference in NLR levels after mechanical support between the “suspected” and the “non-suspected” infection group (Online resource 4).

**Fig. 3:**
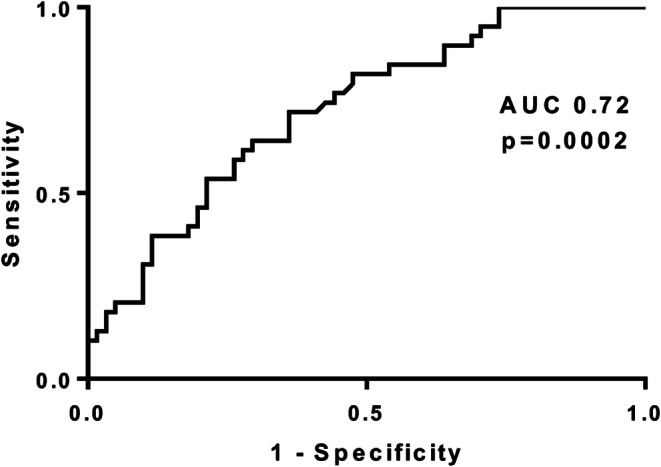
Prognostic value of baseline neutrophil-to-lymphocyte ratio in survival of patients with cardiogenic shock

We performed a stepwise logistic regression analysis which included NLR, age, hemoglobin, creatinine, right atrial pressure, WBC, and RDW. NLR remained an independent predictor of in-hospital mortality in the study population after adjusting for age and hemoglobin (OR 0.913, CI 0.85–0.98) (Table 2). RDW and age also remained independent predictors. Creatinine, right atrial pressure, WBC, and hemoglobin were significant predictors in the univariate analysis but were not significant predictors after adjusting for other variables and were not included in the final model. Finally, NLR had a good discriminatory power as shown in ROC curve of Fig. 3 (AUC 0.72, *p* = 0.0002).

**Table 2:** Predictors of survival in patients with cardiogenic shock

	Unadjusted OR (95%CI)	*p* value	Adjusted OR (95%CI)	*p* value
NLR	0.907 (0.85–0.97)	0.003	0.913 (0.85–0.98)	0.018
Age	0.946 (0.91–0.98)	0.002	0.919 (0.87–0.97)	0.001
RDW	0.730 (0.6–0.9)	0.003	0.707 (0.548–0.913)	0.008
Hemoglobin	1.331 (1.1–1.6)	0.002	---	---
Creatinine	0.55 (0.37–0.84)	0.005	---	---
Right atrial pressure	0.902 (0.84–0.97)	0.006	---	---
WBC	0.919 (0.85–0.99)	0.022	---	---

*WBC* white blood cells, *RDW* red cell distribution width, *NLR* neutrophil-to-lymphocyte ratio

## Discussion

We aimed to investigate the role of systemic inflammation as predictor of outcomes in patients with CS supported by percutaneous assist device. Our retrospective analysis showed that NLR, a marker of systemic inflammation, was higher in patients with advanced stages of CS, suggesting a correlation between inflammation and disease severity. Along these lines, the inflammatory cytokine IL6 was higher in patients with biventricular failure requiring ECMO support compared with patients with isolated left ventricular (LV) failure requiring Impella support. Additionally, NLR at the time of device implantation was lower in the subgroup of survivors and was shown to be an independent predictor of outcomes in patients with CS even after adjustment. Finally, we observed a reduction in IL6 levels after mechanical support associated with survivorship, indicating that reversal of inflammation could serve as an adjuvant therapeutic target for CS treatment.

Several studies have shown a link between inflammation and development of chronic heart failure [2]. While cardiac and systemic inflammation have been used as prognostic markers in chronic HF population, the data in acute HF and CS are scarce and limited mainly to the post-myocardial infarction population [9]. In a meta-analysis of TACTICS-TIMI 18, elevated WBC and C-reactive protein (CRP) at the time of unstable angina/non-ST elevation myocardial infarction (UA/NSTEMI) presentation correlated with higher 6-month mortality [10]. Contrary to TACTICS-TIMI 18 which included hemodynamically stable patients, our study included critically ill patients presenting with CS. In addition, our study population was more diverse, including both patients presenting with post-myocardial infarction (MI) shock and shock in the setting of acute on chronic HF exacerbation. Despite these differences, we were also able to detect lower WBC levels in the subgroup of survivors at the time of presentation suggesting that circulating leukocytes correlate with outcomes in patients with CS.

Inflammatory cytokines have been used as surrogate marker of systemic inflammation and their circulating levels have been associated with the severity and progression of HF [11, 12]. The decision to selectively measure the levels of IL6, TNFa, and IFNg was based on data from our prior study showing that those were among the most prominent circulating cytokines from a panel of 13 proinflammatory cytokines in a chronic HF population supported by durable LVAD [13]. In agreement to our prior chronic HF study, IL6 was the highest detected cytokine in our acute HF population while TNFa and IFNg were detected in very low levels. This discrepancy suggests that despite the important role of inflammation in the pathogenesis of both acute and chronic HF, the biologic significance of each cytokine may differ in those two conditions.

IL6 has been previously identified as predictor of outcomes in patients with septic and CS [14, 15]. In a prospective study of patients with MI complicated by CS, IL6 was an independent early prognostic marker of 30-day mortality [14]. Our study did not have the power to examine the role of IL6 as a predictor of outcomes. However, we report higher IL6 levels in patients undergoing ECMO compared with Impella support, suggesting that patients with worse hemodynamic profile, requiring biventricular support, have higher inflammatory burden compared with patients requiring only LV support. A novel finding of our study is that IL6 levels decrease after mechanical support only in the serum of survivors and continue to rise in the serum of non-survivors. This is different from the findings of Andrie et al., who reported reduction in IL6 after mechanical support, regardless of the outcome. One explanation of this discrepancy could be the use of intra-aortic balloon pump (IABP) as a percutaneous support device which provides less hemodynamic support compared with ECMO and Impella and as a result, it may have less effect in the reversal of systemic inflammatory response.

Animal studies have shown that administration of anti IL6 antibody prior to myocardial infarction reduces the leukocyte and macrophages infiltration and leads to reduced cardiac dilation and improved cardiac function [16]. The effect of anti-IL6 therapies has not been studied in either experimental or clinical studies of cardiogenic shock. However, IL6 blockade in animal models of septic shock has shown significant improvement of survival likely through reduction of systemic inflammation [17]. We hypothesize that extreme cardiogenic shock is also a systemic inflammatory process affecting systemic vascular permeability and end organ function. In our study, IL6 reduction in the serum of survivors suggests that IL6 blockade might serve as a therapeutic target for patients with cardiogenic shock.

While the circulating cytokines could provide a mechanistic insight and serve as potential targets in the treatment of CS, their role as outcome predictors is limited by the fact that they are expensive and difficult to measure. On the contrary, NLR is an easily accessible marker that could serve as a surrogate marker of systemic inflammation. NLR has been found to predict cardiovascular mortality in asymptomatic adults and in patients with acute coronary syndrome [18, 19]. In a recent study of end-stage HF patients, high NLR prior to *durable circulatory support device* implantation was independently associated with worse 90-day outcomes [20]. In our study, higher NLR levels prior to *temporary circulatory support device* implantation correlated with the higher SCAI shock class, indicating a relationship between the severity of CS and the systemic inflammatory burden. These data provide further support to the idea that in addition to interventions that target the cardiac muscle, adjuvant therapies that support the peripheral organs and block the systemic inflammatory response could improve the survival from CS. The median NLR in our population was significantly higher compared with the chronic stable CS patients undergoing LVAD implantation (7.4 vs 4.7). This difference could be explained by the acute inflammatory response that was induced by the acute HF exacerbation leading to CS. Regardless though of the NLR value, it remained an independent predictor of survival even after correcting with established clinical markers of disease severity.

## Conclusion

In summary, our data suggest that there is a correlation between systemic inflammation and severity of CS. NLR is an easily accessible marker of inflammation that could serve as an independent predictor of outcomes in patients presenting with CS. Whether anti-inflammatory therapies targeting specific cytokines and/or inflammatory pathways could improve survival in CS requires further investigation.

### Clinical Significance

Our findings indicate that NLR could be used as a biomarker for the prediction of outcomes in patients presenting with severe cardiogenic shock. Patients that are high risk for poor outcomes, based on their baseline elevated NLR, should be candidates for further escalation of their circulatory support and for earlier transition to a durable device or heart transplantation. In addition, our findings indicate that additional studies are required in order to shed light on the role of anti-inflammatory medications as adjuvant therapies for severe cardiogenic shock.

### Limitations

Despite the relatively large size of our study population, it remains a small retrospective study. The lack of validation cohort is also a major limitation of our study. The cytokine levels were measured in a small subgroup of our patients and we did not have the statistical power to include them in our multivariate analysis. Finally, our study is not designed to investigate whether the activation of inflammatory pathways plays a causative role in the development of CS or just represents an epiphenomenon and a marker of disease severity. Larger, multicenter prospective studies are necessary to confirm our findings and animal studies are necessary to establish causation between inflammation and CS.

## Electronic Supplementary Material

ESM 1 (DOC 621 kb).

## Abbreviations

CS: Cardiogenic shock
ECMO: Extra-corporeal membrane oxygenator
EF: Ejection fraction
HF: Heart failure
IFN: Interferon
IL: Interleukin
LVAD: Left ventricular assist device
NLR: Neutrophil-to-lymphocyte ratio
TNF: Tumor necrosis factor
