# Supplementary material for: Systemic Inflammatory Burden Correlates with Severity and Predicts Outcomes in Patients with Cardiogenic Shock Supported by a Percutaneous Mechanical Assist Device
Source: J Cardiovasc Transl Res. 2020 Oct 19;14(3):476–83. doi: 10.1007/s12265-020-10078-5 (PMC9643251; doi:10.1007/s12265-020-10078-5)
Supplement: Supplementary file 1 — (DOC 621 kb). [file 12265_2020_10078_MOESM1_ESM.doc]

**Supplemental Material**

**Supplemental Results**

**Online resource 1.** Distribution of study population in SCAI shock classes.

**a**

**c**

**b**

**d**

**Online resource 2. (a)** IL6, **(b)** TNFa, **(c)** IFNg and **(d)** NLR levels in patients with cardiogenic shock and ischemic vs non-ischemic cardiomyopathy. Patients with cardiogenic shock and non ischemic cardiomyopathy have higher IL6 levels prior to percutaneous device implantation compared to patients with ischemic cardiomyopathy. There was no difference in TNFa, IFNg and NLR between the two groups.

**Online resource 3.** Leukocyte populations prior to device implantation is survivors and non survivors. WBC: White Blood Cells, Neut: Neutrophils, Lymph: Lymphocytes, Mono: Monocytes, Eos: Eosinophils.

**Online resource 4.** There is no difference in NLR levels either pre or post mechanical support in patients with suspected infection and in patients without suspected infection.
